# The M_1_ muscarinic acetylcholine receptor subtype is important for retinal neuron survival in aging mice

**DOI:** 10.1038/s41598-019-41425-5

**Published:** 2019-03-26

**Authors:** Panagiotis Laspas, Mayagozel B. Zhutdieva, Christoph Brochhausen, Aytan Musayeva, Jenia Kouchek Zadeh, Norbert Pfeiffer, Ning Xia, Huige Li, Juergen Wess, Adrian Gericke

**Affiliations:** 1 Department of Ophthalmology, University Medical Center, Johannes Gutenberg University Mainz, Mainz, Germany; 2 Institute of Pathology, University Medical Center, Johannes Gutenberg University Mainz, Mainz, Germany; 3 Institute of Pharmacology, University Medical Center, Johannes Gutenberg University Mainz, Mainz, Germany; 4 Molecular Signaling Section, Laboratory of Bioorganic Chemistry, National Institute of Diabetes and Digestive and Kidney Diseases, National Institutes of Health, Bethesda, Maryland USA

## Abstract

Muscarinic acetylcholine receptors have been implicated as potential neuroprotective targets for glaucoma. We tested the hypothesis that the lack of a single muscarinic receptor subtype leads to age-dependent neuron reduction in the retinal ganglion cell layer. Mice with targeted disruption of single muscarinic acetylcholine receptor subtype genes (M_1_ to M_5_) and wild-type controls were examined at two age categories, 5 and 15 months, respectively. We found no differences in intraocular pressure between individual mouse groups. Remarkably, in 15-month-old mice devoid of the M_1_ receptor, neuron number in the retinal ganglion cell layer and axon number in the optic nerve were markedly reduced. Moreover, mRNA expression for the prooxidative enzyme, NOX2, was increased, while mRNA expression for the antioxidative enzymes, SOD1, GPx1 and HO-1, was reduced in aged M_1_ receptor-deficient mice compared to age-matched wild-type mice. In line with these findings, the reactive oxygen species level was also elevated in the retinal ganglion cell layer of aged M_1_ receptor-deficient mice. In conclusion, M_1_ receptor deficiency results in retinal ganglion cell loss in aged mice via involvement of oxidative stress. Based on these findings, activation of M_1_ receptor signaling may become therapeutically useful to promote retinal ganglion cell survival.

## Introduction

Glaucoma is a common neurodegenerative eye disease, which causes significant visual impairment at the terminal stage. It has been estimated that almost 80 million people worldwide will have glaucoma in 2020^1,2^. The disease is characterized by a progressive death of retinal ganglion cells (RGCs), specific anatomic alterations of the optic nerve and functional defects in the visual field^3^.

Similar to other neurodegenerative diseases, deficits in natural cellular neuroprotective mechanisms may lead to a loss of neurons in the eye, especially under the presence of trigger factors like elevated intraocular pressure (IOP) and advanced age^4,5^. Cholinergic activation of the muscarinic acetylcholine receptor family, which is composed of five subtypes (M_1_-M_5_), exerts well-known neuroprotective effects in the brain^6^. Acetylcholinesterase inhibitors, which increase cholinergic activity, presently have clinical application in the management of Alzheimer’s and Parkinson’s disease^7,8^. However, their clinical utility is limited because of adverse effects associated with simultaneous activation of more than one muscarinic receptor subtype^9^.

The neuroprotective properties of cholinergic stimulation in the brain are mostly attributed to M_1_ receptor subtype activation. For that reason, new strategies are pursued in the therapy of Alzheimer’s disease to selectively activate the M_1_ receptor. Examples are the development of highly selective agonists for the M_1_ receptor subtype^10^ or the use of positive allosteric modulators, which selectively enhance M_1_ receptor affinity to acetylcholine^11,12^.

All five subtypes of the muscarinic receptor family have been identified in the eye, where they contribute to numerous physiological actions, such as regulation of IOP, pupil size, and ocular growth^13^. In the retina, muscarinic receptors are involved in modulating vasodilatation, cell-to-cell signaling and cell survival^14–16^.

The goal of this study was to examine whether mutations affecting the expression of individual muscarinic receptor subtypes influence neuron survival in the retina. Our hypothesis was that the lack of a single muscarinic receptor subtype leads to age-dependent neuron reduction in the RGC layer. Because of the dearth of highly selective ligands for individual muscarinic receptor subtypes, we used N10 congenic mice of two different age categories, 5 and 15 months, respectively, each lacking one of the five muscarinic receptor subtypes for our studies.

## Materials and Methods

### Animals

All mice were treated in accordance with the EU Directive 2010/63/EU for animal experiments, and all experiments were approved by the Animal Care Committee of Rhineland-Palatinate, Germany. The generation of the different muscarinic receptor mouse knockout (KO) lines (M1R−/−, M2R−/−, M3R−/−, M4R−/−, and M5R−/−) has been described previously^17–21^. The genes that were knocked-out were Chrm1, Chrm2, Chrm3, Chrm4 and Chrm5, respectively. Each KO strain was backcrossed with C57BL/6NTac mice for 10 generations to obtain N10 congenic mice. Polymerase chain reaction (PCR) of DNA isolated from tail biopsies was used to identify the genotype of each animal. All KO mice are complete knockouts with no remaining receptor activity. Inbred C57BL/6NTac mice, throughout referred to as wild-type mice, served as controls.

Mice were housed under standardized conditions with a 12 h light/dark cycle, temperature of 22 ± 2 °C, humidity of 55 ± 10%, and with free access to food and tap water. Experiments were conducted in mice lacking a single muscarinic acetylcholine receptor subtype gene and in wild-type mice at the age of 5 and 15 months, respectively. Altogether there were 12 groups of 8 male mice.

### Measurement of intraocular pressure

A TonoLab rebound tonometer (Bon Optic, Lübeck, Germany) was used for the measurement of IOP. This was conducted non-invasively in restrained conscious mice. Topical anesthesia with proparacaine 0.5% was used directly before each examination. Twelve IOP measurements were taken each time per eye, and the means for both eyes were calculated for each mouse. All measurements were conducted in the evening between 5.00 p.m. and 7.00 p.m.

### Retinal wholemounts and cell counting

**Figure 1:**
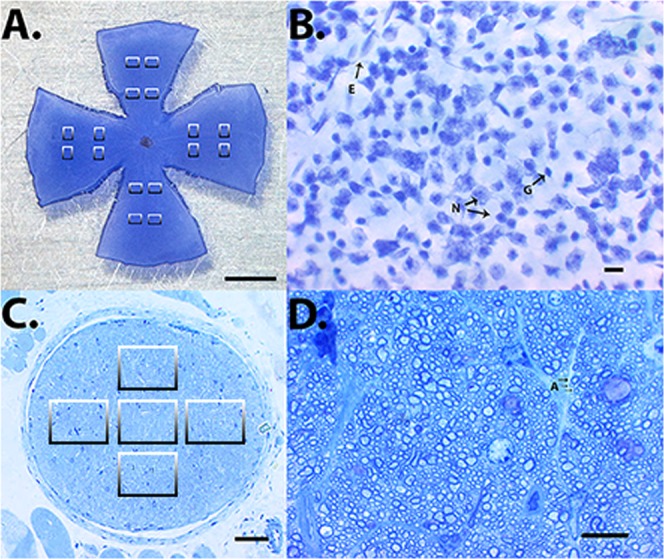
Photographs were taken from 16 pre-defined areas of wholemount stained with cresyl blue (**A**; scale bar 1 mm). In each photograph (**B**; scale bar 10 µm), the individual cell types were identified and separately counted (E: endothelial cell; G: glial cell; N: neuron). In optic nerve cross sections (**C**; scale bar 50 µm) the axon number (A: axon) was counted in 5 microphotographs taken from 5 pre-defined areas (**D**; scale bar 10 µm).

One randomly selected eye per mouse was obtained post mortem and fixed for one hour in 4% of paraformaldehyde (Sigma-Aldrich, Munich, Germany). Next, retinas were isolated in phosphate buffered solution (PBS, Invitrogen, Karlsruhe, Germany), and wholemounts were prepared, put on glass slides and stained with cresyl blue using a standard protocol as described previously^22^. A light microscope (Vanox-T, Olympus, Hamburg, Germany) connected to a Hitachi CCD camera (Hitachi, Düsseldorf, Germany) and equipped with Diskus software (Carl H. Hilgers, Königswinter, Germany) was used for the examination of wholemounts. Sixteen pre-defined areas per wholemount, eight central and eight peripheral, of 150 µm × 200 µm were photographed (Fig. 1A) by a blinded investigator. The proximal border of a central area was localized 0.75 mm from the center of the papilla. This distance corresponded to 5 heights of a photographed area. Each proximal border of a peripheral area was localized 0.75 mm from the distal border of a central photographed area. Thus, the distance from the center of the papilla and the proximal border of a peripheral area was 1.65 mm.

In each photograph (Fig. 1B) cells were counted manually by a blinded investigator using the cell counter plug-in for ImageJ software (NIH, http://rsb.info.nih.gov/ij/). After the photograph was opened in ImageJ under *plugins/analyze*, the *cell counter* tool was initialized. Using this, every cell was manually categorized and marked. Each marked cell was counted and assigned to the predefined category by the software. Cells were subdivided into three types based on morphological criteria of their nucleus (Fig. 1B). Neurons comprised presumptive RGCs with irregular outlines and a prominent nucleolus as well as presumptive displaced amacrine cells with a round or slightly oval, darkly staining nucleus of homogeneous size. Vascular endothelial cells were categorized based on long cell nuclei positioned along a blood vessel. Presumptive glial cells had a small darkly stained nucleus of variable shape. Next, from all 16 counted areas per retina, the average cell density was calculated. After measuring the surface of each wholemount with ImageJ and multiplying it with the cell density of each wholemount, the total number of cells per retina was calculated.

### Optic nerve cross-sections and axon counting

Optic nerves were dissected, placed in fixative solution, postfixed and finally embedded in agar 100 resin. Later, semi-thin cross-sections were cut with an ultramicrotome (Ultracut E, Leica, Bensheim, Germany), placed on conventional glass slides and stained with 1% toluidine blue in 1% sodium borate according to standard protocols (Fig. 1C).

All cross-sections were examined through a light microscope (Vanox-T, Olympus) by a blinded investigator. In photographs of the whole cross-sections, we assessed their entire surface using ImageJ software. Five non-overlapping fields of 80 µm × 60 µm (one central and four in the periphery) were photographed (Hitachi CCD camera) on every cross-section (Fig. 1C). The axons were counted manually on these photographs (Fig. 1D) using ImageJ software. After the photograph was opened in ImageJ under *plugins/analyze*, the *cell counter* tool was initialized. With this tool, each axon was manually marked and counted by the software. The mean axon density was calculated for all 5 counted areas per optic nerve cross section. The total number of axons per optic nerve was determined by multiplying the mean density by the cross sectional area, with was calculated with ImageJ.

### Real-time PCR analysis

We used real-time PCR to determine the expression of different genes in the retina of aged wild-type and M1R−/− mice. Messenger RNA of prooxidative redox enzymes (nicotinamide adenine dinucleotide phosphate oxidases 1, 2, 4 - NOX1, NOX2, NOX4), of antioxidative redox enzymes (superoxide dismutase 1, 2, 3 - SOD1, SOD2, SOD3, glutathione peroxidase-1 - GPx1, heme oxygenese-1 - HO-1, catalase) and of intracellular regulatory proteins involved in aging (Sirtuin 1 - SIRT1, Sirtuin 6 - SIRT6, forkhead box protein O1 - FOXO1) was quantified.

After mice had been killed by CO_2_ inhalation, the eyes were immediately removed and placed in ice-cold phosphate buffered solution (PBS, Invitrogen, Karlsruhe, Germany). The retina was isolated under a dissecting microscope by using fine-point tweezers and microscissors, then put into a 1.5-ml tube and immediately frozen in liquid nitrogen. Subsequently, tissue samples were homogenized (FastPrep; MP Biomedicals, Illkirch, France).

Gene expression was measured by SYBR Green based quantitative real time RT-PCR (qPCR), as previously described^23^. RNA was isolated using peqGOLD TriFast™ (PEQLAB) and cDNA was generated with the High Capacity cDNA Reverse Transcription Kit (Applied Biosystems, Darmstadt, Germany). Quantitative real time RT-PCR (qPCR) reactions were performed on a StepOnePlus™ Real-Time PCR System (Applied Biosystems) using SYBR® Green JumpStart™ Taq ReadyMix™ (Sigma-Aldrich, Munich, Germany) and 20 ng cDNA. Relative mRNA levels of target genes were quantified using comparative threshold (CT) normalized to housekeeping gene TATA-binding protein (TBP). The qPCR primer sequences are shown in Table 1.

**Table 1:** Primer sequences for qPCR of the genes examined in aged wild-type and M1R−/− mice.

TBP forward	CTT CGT GCA AGA AAT GCT GAA T
TBP reverse	CAG TTG TCC GTG GCT CTC TTA TT
NOX1 forward	GGA GGA ATT AGG CAA AAT GGA TT
NOX1 reverse	GCT GCA TGA CCA GCA ATG TT
NOX2 forward	CCA ACT GGG ATA ACG AGT TCA
NOX2 reverse	GAG AGT TTC AGC CAA GGC TTC
NOX4 forward	TGT AAC AGA GGG AAA ACA GTT GGA
NOX4 reverse	GTT CCG GTT ACT CAA ACT ATG AAG AGT
SOD1 forward	CCA GTG CAG GAC CTC ATT TTA AT
SOD1 reverse	TCT CCA ACA TGC CTC TCT TCA TC
SOD2 forward	GCT CTG GCC AAG GGA GAT G
SOD2 reverse	TGT CCC CCA CCA TTG AAC TT
SOD3 forward	TTC TTG TTC TAC GGC TTG CTA CTG
SOD3 reverse	AGC TGG ACT CCC CTG GAT TT
GPx1 forward	CCT TGC CAA CAC CCA GTG A
GPx1 reverse	CCG GAG ACC AAA TGA TGT ACT TG
HO-1 forward	GGT GAT GCT GAC AGA GGA ACA C
HO-1 reverse	TAG CAG GCC TCT GAC GAA GTG
Catalase forward	CAA GTA CAA CGC TGA GAA GCC TAA G
Catalase reverse	CCC TTC GCA GCC ATG TG
SIRT1 forward	GCC AAA CTT TGT TGT AAC CCT GTA
SIRT1 reverse	TGG TGG CAA CTC TGA TAA ATG AA
SIRT6 forward	TGG CCC CCA AGT TTG ACA
SIRT6 reverse	GCT GAA CCA GGG CCA TGT
FOXO1 forward	GCG GGC TGG AAG AAT TCA A
FOXO1 reverse	TTC CTT CAT TCT GCA CTC GAA TAA

### DHE Staining

The oxidative fluorescent dye, dihydroethidium (DHE), dihydroethidium (DHE) was utilized to determine reactive oxygen species ROS levels *in situ* as described previously^24^. After mice had been sacrificed and their eyes harvested, 10 µm frozen cross-sections were prepared. After thawing, the tissue sections were immediately incubated with 1 µmol/l dihydroethidium. Dihydroethidium is cell permeable and reacts with superoxide to form ethidium, which in turn intercalates in the deoxyribo nucleic acid, thereby exhibiting a red fluorescence. Photographs of retinal cross-sections were taken using a fluorescent microscope at an excitation wavelength of 520 nm and an emission wavelength of 610 nm. The intensity of the staining was measured in individual cell layers of the retina by using ImageJ software (NIH, http://rsb.info.nih.gov/ij/).

### Statistical analysis

Data are presented as mean ± SD unless other stated, and n represents the number of mice (eyes) per group. For comparison of IOP, cell and axon number between young and old mice of the same genetic background, an unpaired t test was used. Different genotypes of the same age category were compared by one-way ANOVA and a Tukey’s post hoc test. Messenger RNA expression levels (ΔCT values) were compared using an unpaired t test and are presented as relative units to wild-type controls. DHE staining intensity was compared using the Mann-Whitney U test and presented as box plots as relative units to wild-type controls. A value of P < 0.05 was defined as significant.

## Results

### Intraocular pressure

**Figure 2:**
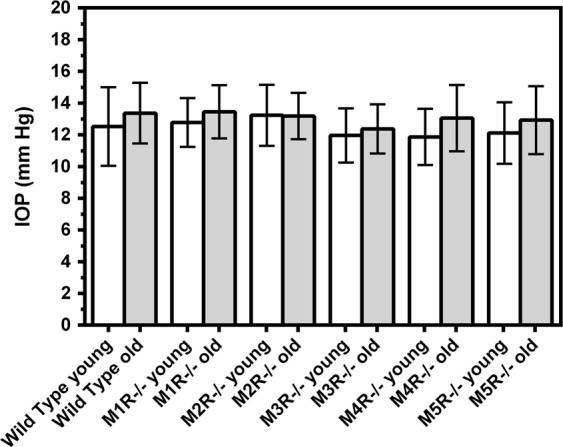
Intraocular pressure (IOP) did not differ between young (5 months) and old (15 months) mice in any of the mouse groups. Also, no differences in IOP were seen between individual mouse genotypes of the same age category. Data are presented as mean ± SD (n = 8 per age category and genotype).

No significant differences in IOP were found between young and old mice of the same genetic background well as between individual mouse genotypes of the same age category (Fig. 2). The mean IOP in individual mouse groups was (5 versus 15 months): Wild-type 12.53 ± 2.479 versus 13.36 ± 1.914, p = 0.46; M1R−/− 12.77 ± 1.536 versus 13.45 ± 1.679, p = 0.41; M2R−/− 13.23 ± 1.924 versus 13.18 ± 1.464, p = 0.96; M3R−/− 11.96 ± 1.712 versus 12.37 ± 1.546, p = 0.62; M4R−/− 11.86 ± 1.774 versus 13.05 ± 2.087, p = 0.24; M5R−/− 12.11 ± 1.938 versus 12.92 ± 2.139, p = 0.44.

### Number of cells in the RGC layer

**Figure 3:**
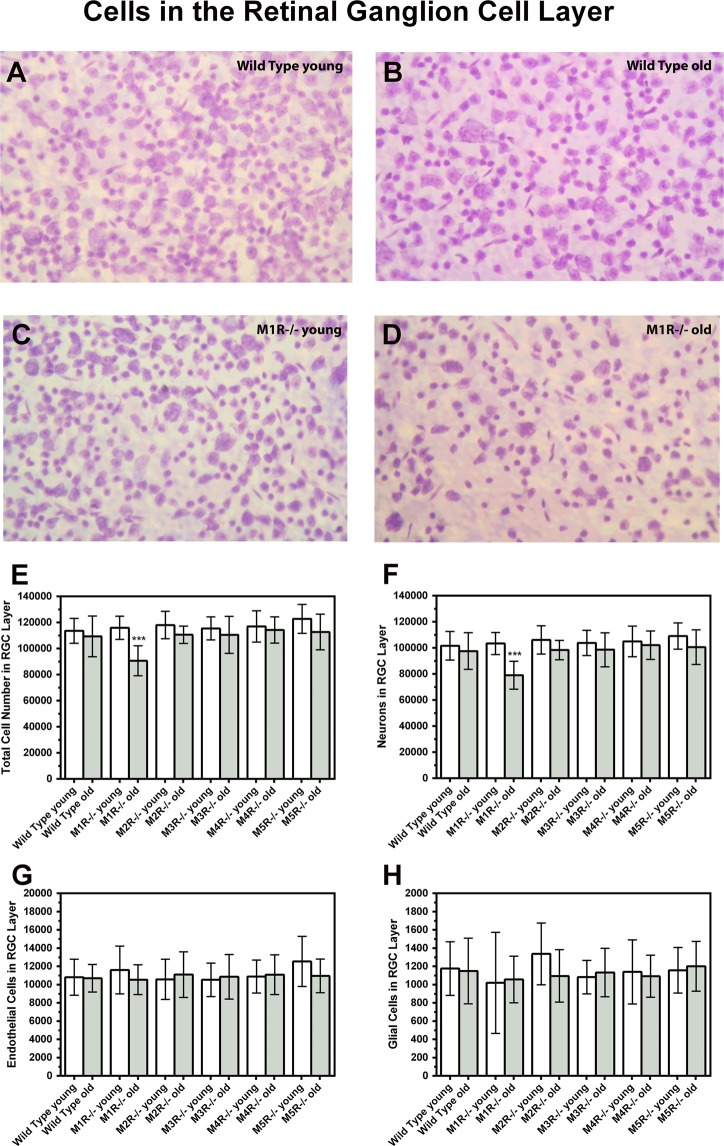
Example photographs taken from retina wholemounts of wild-type and M1R−/− mice at both age categories (**A**–**D**; scale bars 10 µm). Total number of cells (**E**), of neurons (**F**), endothelial cells (**G**) and glia cells (**H**) in the RGC layer. The total number of cells and number of neurons in the RGC layer was found lower in old M1R−/− mice than in young mice of the same genetic background (***P < 0.001).

The total number of cells in the RGC layer did not differ between young and old mice of all genotypes except M1R−/− mice, where the cell number was markedly reduced in aged mice (Fig. 3E). The total cell number in the individual mouse groups was (5 versus 15 months): Wild-type 113598 ± 9543 versus 109390 ± 15565, p = 0.53; M1R−/− 115981 ± 8897 versus 90625 ± 11414, ***p = 0.0002; M2R−/− 118012 ± 10529 versus 110575 ± 6623, p = 0.11; M3R−/− 115438 ± 8833 versus 110539 ± 14156, p = 0.42; M4R−/− 117008 ± 12050 versus 114250 ± 10086, p = 0.63; M5R−/− 122780 ± 11083 versus 112.747 ± 13697, p = 0.13. The total cell number in the RGC layer was also reduced in 15 month-old M1R−/− mice compared to all other genotypes of the same age category (*p < 0.05).

Looking at the subpopulations of cells, we found that 15-month-old M1R−/− mice had a markedly reduced number of neurons in the RGC layer compared to 5-month-old M1R−/− mice (Fig. 3F). In contrast, there were no age-dependent differences in neuron number between old and young mice of the other genotypes. The number of neurons in the individual mouse groups was (5 versus 15 months): Wild-type 101613 ± 10981 versus 97549 ± 14117, p = 0.53; M1R−/− 103362 ± 8438 versus 79028 ± 10752, ***p = 0.0002; M2R−/− 106103 ± 10793 versus 98382 ± 7456, p = 0.12; M3R−/− 103833 ± 9660 versus 98552 ± 13045, p = 0.37; M4R−/− 104981 ± 11715 versus 102071 ± 10941, p = 0.62; M5R−/− 109084 ± 10021 versus 100592 ± 13310, p = 0.17. Neuron number in the RGC layer was also reduced in 15 month-old M1R−/− mice compared to all other genotypes of the same age category (*p < 0.05).

The subpopulation of endothelial cells localized in the RGC layer did not differ in any of the mouse genotypes (Fig. 3G). The number of endothelial cells in the individual mouse groups was (5 versus 15 months): Wild-type 10809 ± 1971 versus 10692 ± 1516, p = 0.90; M1R−/− 11600 ± 2619 versus 10541 ± 1623, p = 0.35; M2R−/− 10573 ± 2198 versus 11098 ± 2501, p = 0.66; M3R−/− 10523 ± 1831 versus 10855 ± 2445, p = 0.76; M4R−/− 10888 ± 1804 versus 11087 ± 2175, p = 0.84; M5R−/− 12539 ± 2753 versus 10954 ± 1853, p = 0.20.

Also, the subpopulation of glial cells localized in the RGC layer did not differ in any of the mouse genotypes (Fig. 3H). The number of glial cells in the individual mouse groups was (5 versus 15 months): Wild-type 1176 ± 293 versus 1149 ± 359, p = 0.87; M1R−/− 1019 ± 554 versus 1056 ± 256, p = 0.87; M2R−/− 1337 ± 339 versus 1095 ± 288, p = 0.15; M3R−/− 1082 ± 183 versus 1133 ± 265, p = 0.67; M4R−/− 1140 ± 352 versus 1092 ± 230, p = 0.75; M5R−/− 1157 ± 250 versus 1201 ± 273; p = 0.74.

### Number of axons in optic nerve cross-sections

**Figure 4:**
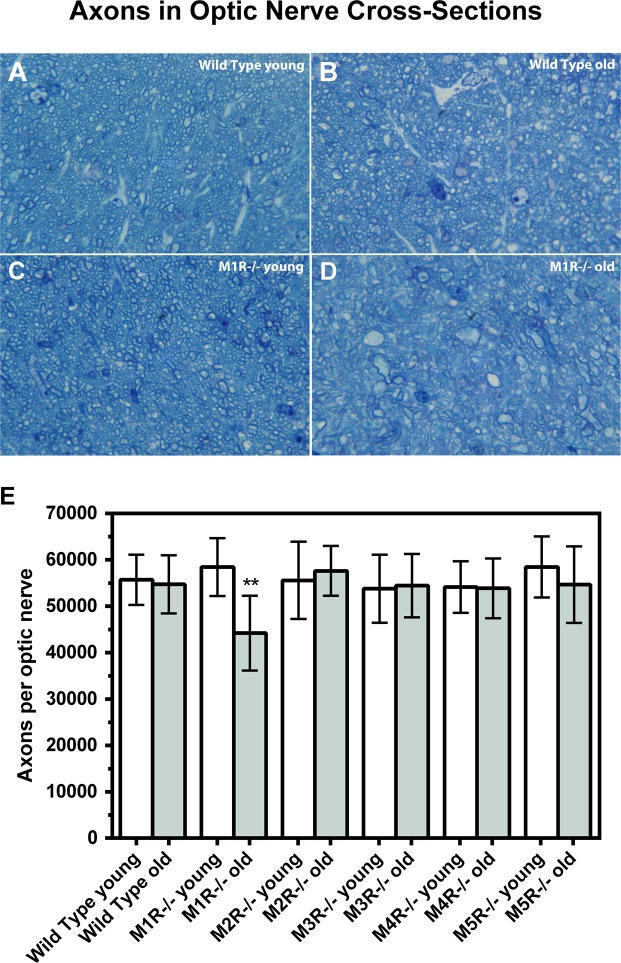
Example of photographs taken from optic nerve cross-sections of wild-type and M1R−/− mice. at both age categories (**A**–**D**; scale bars 10 µm). The number of optic nerve axons was lower in old M1R−/− mice compared to younger mice of the same genetic background (D, **p < 0.01).

The number of axons in the optic nerve, which represent the number of RGC, was markedly reduced in 15-month-old compared to 5-month-old M1R−/− mice (Fig. 4E). In contrast, no differences in axon number were found between young and old mice of all other genotypes. The whole axon number in individual mouse groups was (5 versus 15 months): Wild-type 55702 ± 5429 and 54721 ± 6269, p = 0.74; M1R−/− 58455 ± 6252 versus 44210 ± 8069, **p = 0.0015; M2R−/− 55585 ± 8346 versus 57636 ± 5362, p = 0.57; M3R−/− 53789 ± 7359 versus 54443 ± 6838, p = 0.86; M4R−/− 54131 ± 5562 and 53871 ± 6423, p = 0.93; M5R−/− 58457 ± 6579 versus 54659 ± 8236, p = 0.33. The axon number in the optic nerve was also reduced in 15 month-old M1R−/− mice compared to wild-type, M2R−/− and M5R−/− mice of the same age category (*p < 0.05).

### Messenger RNA expression

**Figure 5:**
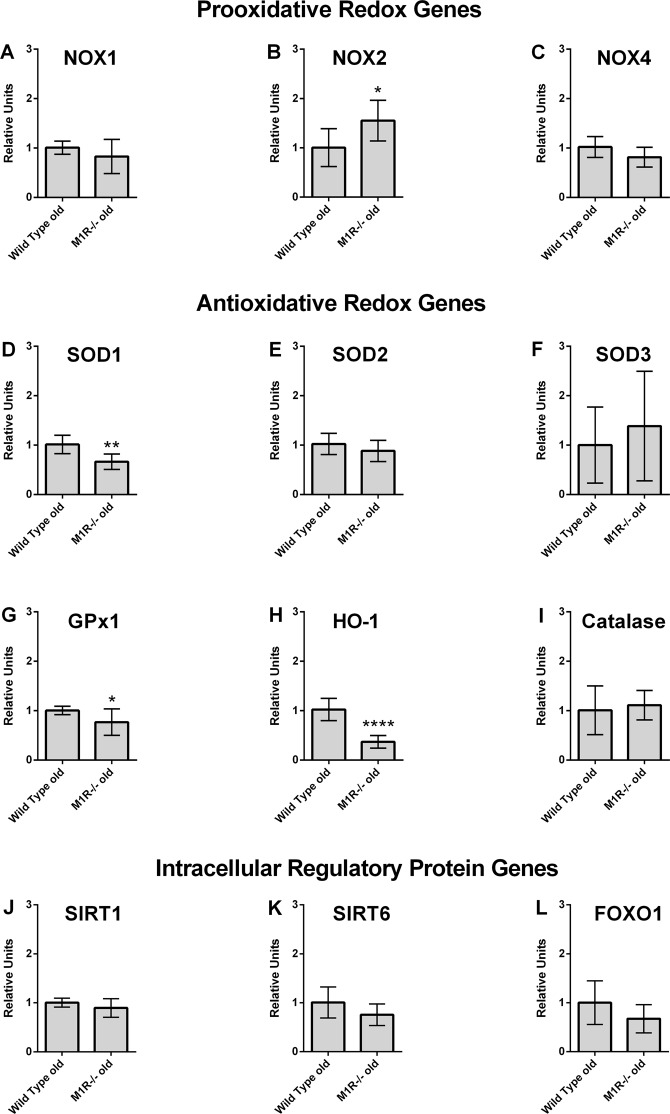
Messenger RNA expression of prooxidative genes (NOX1, NOX2, NOX4), antioxidative genes (SOD1, SOD2, SOD3, GPx1, HO-1, Catalase) and of genes coding for intracellular regulatory proteins involved in aging (SIRT1, SIRT6, FOXO1) in aged wild-type and M1R−/− mice. Expression of NOX2 was significantly increased in aged M1R−/− mice. In contrast, the antioxidative redox genes, SOD1, GPx1 and HO-1, showed reduced expression levels in aged M1R−/− mice. Data are presented as mean ± SD (n = 8 per genotype; *p < 0.05; **p < 0.01; ****p < 0.0001).

Expression of the genes examined is presented in Fig. 5. Among the three prooxidative NOX isoforms (NOX1, NOX2, NOX4) that have previously been shown to be modified in different pathological conditions of the retina, NOX2 was found to be higher expressed in the retina of aged M1R−/− mice compared to wild-type mice (*p = 0.0157). Interestingly, several antioxidative redox genes, including SOD1 (**p = 0.0011), GPx1 (*p = 0.0324) and HO-1 (****p < 0.0001), displayed lower mRNA expression levels in M1R−/− mice than in wild-type mice. The expression levels of genes coding for intracellular regulatory proteins involved in aging, SIRT1, SIRT6 and FOXO1, did not differ between aged wild-type and M1R−/− mice.

### ROS levels in the retina

**Figure 6:**
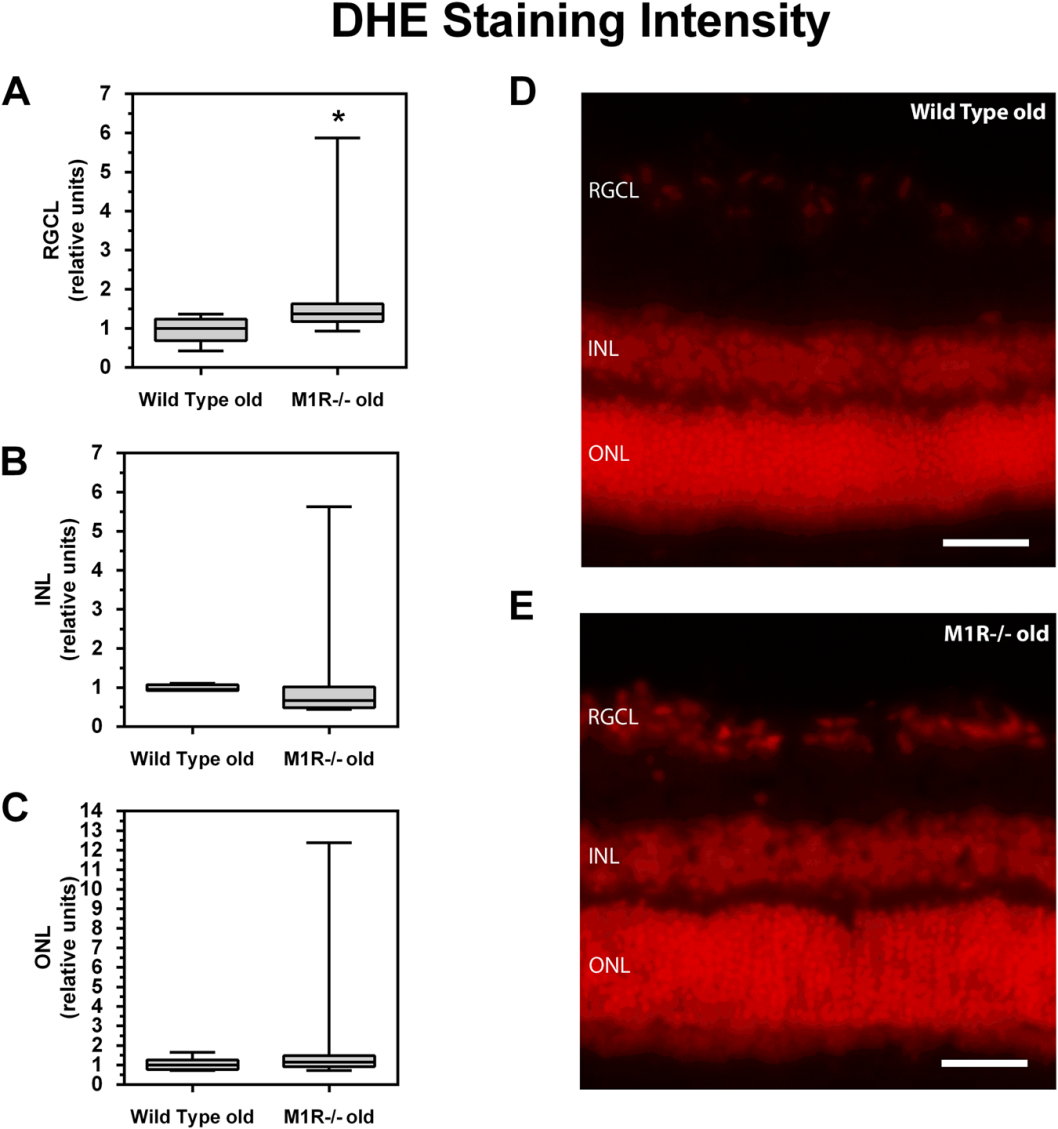
DHE staining intensity in the retinal ganglion cell layer (RGCL, **A**), the inner nuclear layer (INL, **B**) and the outer nuclear layer (ONL, **C**) in aged wild-type and M1R−/− mice. DHE staining intensity was markedly elevated in the RGCL of aged M1R−/− mice (*P < 0.05) suggesting higher ROS levels. Pictures of DHE-stained retinal cryosections for aged wild-type (**D**; scale bar 50 μm) and M1R−/− mice (**E**; scale bar 50 μm).

The DHE staining intensity was markedly higher in the RGC layer of aged M1R−/− mice compared to age-matched wild-type mice (*p = 0.0148; Fig. 6A), indicative of increased ROS levels. In contrast, no significant differences in staining intensity between the two mouse genotypes were found in the inner nuclear layer (Fig. 6B) and the outer nuclear layer (Fig. 6C).

## Discussion

There are two major new findings in the present study. First, the lack of the M_1_ receptor age-dependently reduced survival of neurons in the RGC layer and of optic nerve axons, which represent the axons of RGC. Because the IOP was not affected by the lack of any of the five muscarinic receptor subtypes at any age, it is unlikely that IOP contributed to the neuron loss of aged M1R−/− mice. Second, mRNA expression for the prooxidative redox enzyme, NOX2, was increased in aged M1R−/− mice while mRNA expression for the antioxidative redox enzymes, SOD1, GPx1 and HO-1, was reduced. Moreover, the ROS level was increased in the RGC layer of aged M1R−/− mice compared to wild-type of the same age, indicating that aged M1R−/− mice had a reduced antioxidative capacity. This is the first study based on gene targeting technology supporting the concept that the neuroprotective effect of the cholinergic system in the RGC layer is mediated by the M_1_ muscarinic receptor subtype.

Previous studies suggested that cholinergic muscarinic receptor activation exerts neuroprotective effects in retinal neurons. For example, activation of muscarinic receptors by carbachol, an acetylcholine analogue, promoted cell survival in rat RGC cultures. This effect was diminished by telenzepine, a M_1_-preferring muscarinic receptor blocker, suggesting that the M_1_ receptor might have mediated the neuroprotective effect observed previously^25^. Pilocarpine, a well-known muscarinic receptor agonist, was found to act protective against glutamate-induced apoptosis in rat retinal cell cultures. This effect was also attributed to the M_1_ receptor, since it was blocked by pirenzepine, a M_1_ receptor-preferring antagonist^26^. Moreover, in an i*n vivo* study, the acetylcholinesterase inhibitor, galantamine, exerted neuroprotective actions in the rat retina, which were blocked by M_1_-preferring muscarinic receptor antagonists^27^. In all these studies, the neuroprotective effect induced by cholinergic activation was reversed by pharmacological blockade of the M_1_ receptor, suggesting its neuroprotective action. However, the selectivity of muscarinic receptor agonists and antagonists utilized in the above cited studies is limited, since the receptor subtypes have only minor differences in their orthosteric binding sites^28^. For example, pirenzepine has only a ten-fold higher affinity to the M_1_ receptor than to all other muscarinic receptor subtypes^29^. Telenzepine shows a similar affinity profile^30^. Thus, a possible neuroprotective effect mediated via other muscarinic receptor subtypes cannot be excluded when using classical pharmacological ligands.

In order to circumvent this problem, we used mice with targeted disruption of single muscarinic receptor subtype genes (M_1_−M_5_) in the present study. We hypothesized, that if a specific muscarinic receptor subtype was responsible for the neuroprotective cholinergic effects in the retina, its total absence in gene-targeted mice would make neurons more prone to cell death.

In a previous study from our laboratory conducted on 5 month-old M1R−/− mice of a mixed genetic background, we found that neuron number in the RGC layer and optic nerve axon number were not affected by M_1_ receptor deficiency^31^. One possible explanation for these findings was that the M1R−/− mice used in the previous study might have been too young to develop neuronal loss. Another explanation was that other muscarinic receptor subtypes might have been responsible for the neuroprotective effects that had previously been attributed to the M_1_ receptor. Hence, in the present study, we examined the potential role of all five muscarinic receptor subtypes on neuroprotection in the RGC layer. Importantly, the animals used in this study were N10 congenic, which makes it much less likely that strain differences may have affected neuron survival. We additionally examined animals at advanced age, which is a crucial risk factor for glaucoma.

Of note, M1R−/− mice had significantly fewer neurons in the RGC layer and axons in the optic nerve at the age of 15 months compared to the age of 5 months. All other genotypes including wild-type mice had similar neuron numbers at young and old age. In previous studies that examined RGCs in mice at different ages also no signs of RGC loss were found in wild-type mice of the C57BL/6 background until the age of 14 months^32^ or even 16 to 23 months^33^.

The neuron loss in M1R−/− mice is unlikely to be caused by an increased IOP, the major risk factor for glaucoma, because no differences in IOP were found between young and aged M1R−/− mice as well as between individual mouse genotypes. Although activation of muscarinic receptors by agonists, such as pilocarpine or carbachol was shown to reduce IOP in humans and mice^34–36^, it does not necessarily mean that the lack or blockade of muscarinic receptors evokes an opposite effect, at least in eyes without preexisting anterior eye abnormalities. For example, no marked impact on IOP has been shown for the muscarinic receptor antagonist, atropine, when applied in humans on a long-term basis^37,38^. Hence, it is possible that muscarinic receptors only affect the IOP level when activated.

The present study supports previous reports suggesting that muscarinic receptor agonists exert neuroprotective effects in the eye not only via IOP reduction, but also by direct action on retinal neurons^27,39^. In the retina, cholinergic amacrine cells are the source of the physiological muscarinic receptor agonist, acetylcholine, which was shown to transmit cholinergic signals to RGCs via muscarinic and nicotinic receptors^40–43^. However, although multiple muscarinic receptor subtypes, including the M_1_ receptor, were detected on RGCs, amacrine cells and bipolar cells, the specific role of the individual subtypes in retinal cell-to-cell signaling is still poorly understood^31,42^. Overlapping receptor subtype expression patterns in individual cell types, limited selectivity of pharmacological ligands and low specificity of antibodies for individual muscarinic receptor subtypes are obstacles in this regard^28,44,45^. Genetically modified animals lacking individual muscarinic receptor subtypes may be useful to circumvent these problems.

In the present study, we assessed neuron loss using two approaches. In the retina, we counted cells in the RGC layer based on morphological criteria of cell nuclei stained with cresyl blue. The staining method has the advantage of being fast and inexpensive, yielding simultaneous information on multiple cell types, being independent on axonal transport properties compared to retrograde labeling techniques, and of circumventing problems of antibody specificity to retinal cells^46–48^. However, this method has also some weaknesses. First, it does not allow to clearly distinguish between RGCs and other neurons, like displaced amacrine cells, because of some overlap in nuclear size and shape^49,50^. Second, the counting method has not been fully validated and, thus, the calculated cell number may vary depending on the evaluator. Third, it should be considered that the presented mathematical average of the sampled areas multiplied by retinal area may result in calculated cell numbers that are skewed from the actual number, because cell density in the RGC layer markedly differs between the center and the periphery of the retina^49^.

In order to specify the number of RGCs, we additionally calculated axons of RGCs in optic nerve cross sections. The axon number as a percentage of neurons in the RGC layer was between 51,6% and 56,6% among all our mouse groups, which is similar to other studies in mice and can be explained by the fact that only around half of the neurons in the RGC layer are actually RGCs^51^. Remarkably, the axon number was markedly reduced in aged M1R−/− mice supporting the notion that RGC survival is compromised with aging when the M_1_ receptor is lacking. However, the relation of axons in optic nerve cross sections to neurons in the RGC layer did not change with age in any of the mouse genotypes, including M1R−/− mice, suggesting that neuron loss in the RGC layer observed in M1R−/− was not selectively affecting RGCs.

Muscarinic receptor-independent gene mutations of the knockout mice that may affect neuron survival in the retina should also be considered. Notably, C57BL/6N substrains were shown to harbor the recessive single base pair mutation, rd8, in the Crb1 gene, which causes retinal degeneration 8, a mild form of retinal degeneration characterized by focal external limiting membrane and photoreceptor defects^52,53^. The onset of the phenotype appears to be between 2 and 6 weeks of age^54^. As opposed to any of the five muscarinic receptor subtype genes, the rd8 mutation is located on chromosome 1, and is not linked with any of the muscarinic receptor mutations. Moreover, rd8 mutation has not been associated with neuron loss in the RGC layer. Thus, it is very unlikely that this mutation is responsible for the neuron loss that we observed in aged M1R−/− mice.

The age-dependent neuron loss in the retina and optic nerve of M1R−/− mice seems highly relevant regarding the pathophysiology of normal tension glaucoma. Most known mouse models of normal tension glaucoma, like excitatory amino-acid carrier 1 (EAAC1), glutamate/aspartate transporter (GLAST) and superoxide dismutase 1 (SOD1) knockout mice are actually models of increased oxidative stress^55–57^. Remarkably, we found that mRNA expression of the prooxidative NOX2 was increased in aged M1R−/− mice. Previous studies reported an increased NOX2 expression to be involved in the pathophysiology of various retinal diseases. For example, NOX2-generated ROS were shown to contribute to retinal vascular injury and to acceleration of vascular endothelial cell senescence in diabetic mouse retinas^58,59^. Moreover, a correlation between elevated retinal apoptotic markers and increased NOX2 expression has been reported in diabetic mice^60^. Also, glucotoxic and lipotoxic insults were shown to promote ROS production in retinal vascular endothelial cells via activation of NOX2^61^. In a mouse model of oxygen-induced retinopathy, NOX2-generated ROS facilitated neovascularization^62^. NOX2 was also shown to promote apoptosis in the retina after ischemic injury^63^. Moreover, NOX2 activity was suggested to play a primary role in acute and chronic conditions associated with retinal vascular inflammatory reactions^64^. In addition to an increased NOX2 expression, we found that mRNA levels of the antioxidative enzymes, SOD1, HO-1 and GPx1, were reduced in aged M1R−/− mice. Importantly, each of the three enzymes has previously been reported to play a neuroprotective role in the retina. For example, the lack of SOD1 resulted in an increased functional impairment of retinal neurons following exposure to paraquat and hyperoxia^65^. Moreover, the lack of SOD1 caused neurodegeneration in the retina of respective KO mice^66^. The lack of SOD1 is correlated with an accelerated aging phenotype. Conversely, an overexpression of SOD1 has been found protective against changes seen in diabetic retinopathy^67^. Our findings are also in line with a study reporting that 24-week-old SOD1 KO mice showed morphological alterations similar to those observed in normal tension glaucoma^57^. In the same study, SOD1 serum levels were found to be reduced in patients with normal tension glaucoma compared to healthy controls^57^. Apart from SOD1, also HO-1 and GPx1 have been proposed to play a protective role against ocular diseases like diabetic and light-induced retinopathies^68,69^. With regard to neuronal degeneration, it has been demonstrated that lower levels of HO-1 increased susceptibility of RGCs to apoptosis^70^. Moreover, reduced levels or the complete lack of GPx1 were shown to enhance the functional, cellular and vascular damage in the retina induced by light^71^, hypoxia^72^ and retinopathy of prematurity^73^.

Taken together, our results suggest that in addition to increased NOX2 mRNA expression levels, aged M1R−/− mice have also a reduced antioxidative capacity in the retina due to downregulation of various antioxidative enzymes, such as SOD1, HO-1 and GPx1, whose lack has been linked to retinal neuron damage. The increased ROS levels that we found in the RGC layer of aged M1R−/− mice support our mRNA expression data. Our findings provide the first direct evidence that the lack of the M_1_ receptor leads to accelerated RGC loss in mice via an increase of oxidative stress in the retina. From a clinical point of view, M_1_ receptor agonists may become useful to treat neurodegenerative ocular diseases, such as glaucoma. Moreover, it would be important to investigate whether certain polymorphisms of the M_1_ receptor gene are associated with glaucoma development in humans.

## Data Availability

Data are available after request to the author.
